# Comparative Glycopeptide Analysis for Protein Glycosylation by Liquid Chromatography and Tandem Mass Spectrometry: Variation in Glycosylation Patterns of Site-Directed Mutagenized Glycoprotein

**DOI:** 10.1155/2018/8605021

**Published:** 2018-09-02

**Authors:** Young Hye Hahm, Sung Ho Hahm, Hyoun Young Jo, Yeong Hee Ahn

**Affiliations:** ^1^Rophibio Inc., Cheongju 28160, Republic of Korea; ^2^Department of Biomedical Science, Cheongju University, Cheongju 28160, Republic of Korea

## Abstract

Glycosylation is one of the most important posttranslational modifications for proteins, including therapeutic antibodies, and greatly influences protein physiochemical properties. In this study, glycopeptide mapping of a reference and biosimilar recombinant antibodies (rAbs) was performed using liquid chromatography-electrospray ionization tandem mass spectrometry (LC-ESI-MS/MS) and an automated Glycoproteome Analyzer (GPA) algorithm. The tandem mass analyses for the reference and biosimilar samples indicate that this approach proves to be highly efficient in reproducing consistent analytical results and discovering the implications of different rAb production methods on glycosylation patterns. Furthermore, the comparative analysis of a mutagenized rAb glycoprotein proved that a single amino acid mutation in the Fc portion of the antibody molecule caused increased variations in glycosylation patterns. These variations were also detected by the mass spectrometry method efficiently. This mapping method, focusing on precise glycopeptide identification and comparison for the identified glycoforms, can be useful in differentiating aberrant glycosylation in biosimilar rAb products.

## 1. Introduction

Glycosylation involves the covalent attachment of glycans to specific amino acid residues on protein and is one of the important posttranslational protein modification processes [1]. Glycosylation alters the properties of proteins including pharmacokinetics, effector functions, solubility, and stability [2]. Aberrant glycosylation in glycoproteins has been related to the occurrence and progression of certain diseases [3]. Careful observation of protein glycosylation is crucial for the development of stable and effective drugs. Furthermore, they are crucial in comparing biosimilar products to reference drugs, as mandated by regulatory agencies [4]. Changes in manufacturing process conditions for biologics, such as process optimization, scale-up production, and site changes, may impact glycosylation patterns of the resulting recombinant antibody (rAb) [5, 6]. As such, glycosylation is considered a critical quality attribute (CQA) for rAb therapeutics as it has the potential to determine whether the biosimilar candidate is highly similar to the reference drug. The glycosylation pattern of a rAb affects a wide spectrum of biological processes. Therefore, consistent glycosylation is necessary to prove the drug's ability to maintain safety and efficacy [7].

Mass spectrometry is a core technology in the field of proteomics with high-throughput performance and accurate digitalized informatics [8]. One particular mass analysis technique is matrix assisted laser desorption/ionization mass spectrometry (MALDI-MS) equipped with time-of-flight (TOF) analyzer [9–12]. Another widely used method in mass spectrometry is liquid chromatography-electrospray ionization tandem mass spectrometry (LC-ESI-MS/MS) [13–17]. LC-ESI-MS/MS is a robust, high-throughput method in identifying and quantifying glycopeptides in enzymatic digests from various proteomic samples. High-throughput ESI-MS/MS for glycopeptides, initially separated by LC, allows for the identification and quantification of all detectable features, which, in turn, provides a more detailed account of protein glycosylation patterns. Data analysis for large amounts of raw mass data resulting from LC-ESI-MS/MS for the glycoproteome is a challenging task; therefore, various search engines have been developed such as Glycomaster DB [18], Byonic [19], MAGIC [20], and Glycoproteome Analyzer (GPA) [21]. GPA is capable of identifying site-specific N-glycopeptides efficiently and features the use of 3-top monoisotopic mass peak intensity of glycopeptides [21, 22]. The high-speed mapping of glycopeptides using GPA has proven to display analytical efficiency with a false display rate (FDR) ≤1%.

Here, we have introduced a practical method for mapping and comparing the glycosylation patterns in rAb glycoproteins, in which glycopeptide samples prepared by in-solution or in-gel protein digestion are analyzed by LC-ESI-MS-MS. The developed method is applied for comparative analysis of glycosylation patterns of biosimilar rAbs as well as a mutagenized rAb glycoprotein.

## 2. Experimental

### 2.1. Reagents and Chemicals

The reference rAb glycoprotein (Adalimumab, commercially known as Humira) was obtained from G Sam Hospital (Gyeonggi-do, Korea). HiTrap Mabselect SuRe columns were purchased from GE Healthcare and C_18_ trap column was purchased from Harvard Apparatus (Holliston, MA USA). Trypsin for protein digestion was obtained from Promega (Madison, WI, USA). 1,4-dithiothreitol (DTT), iodoacetamide (IAA), trifluoroacetic acid (TFA), and formic acid (FA) were purchased from Sigma Aldrich (St. Louis, MO, USA). HPLC-grade water and acetonitrile (ACN) were purchased from J.T. Baker (Phillipsburg, NJ, USA). CHO-k1 cells were purchased from ATCC (Manassas, VA, USA) and ExpiCHO-s cells were purchased from Thermo Fisher Scientific (Waltham, MA, USA).

### 2.2. Expression Vector for the Biosimilar

The light chain and heavy chain genes for biosimilar rAb were synthesized (Genscript, Piscataway, NJ, USA) and cloned into a custom expression vector, pCPp2-CMV. The expression vector contains both the heavy chain and the light chain genes for the biosimilar rAb controlled by the human CMV promoter, respectively. The vector also contains puromycin-resistance gene as a selectable marker under SV40 promoter.

### 2.3. Transient Expression of Biosimilar rAbs

For the transient expression of biosimilar rAbs, ExpiCHO-s cells were transfected with the expression vector and maintained in fed-batch culture following manufacturer's protocol for max titer. Briefly, 50 ml of Expi-CHO-s cells cultured in ExpiCHO culture medium in a 250 shaker flask was transfected with 50 *μ*g of the expression vector DNA using Expifectamine CHO reagent. Transfected cells were maintained on shaker at 32°C in a CO_2_ incubator and ExpiCHO enhancer and feed were added as recommended. Cells were monitored every 24 hours for the cell growth and viability. Cells were harvested 7-10 days after transfection before cell viability drops below 75%. Cell supernatant was collected by centrifugation at 3,000 g for 30 min and filtered through a vacuum filtration unit (0.22 um). Filtered cell supernatant was subjected to purification for biosimilar rAbs by protein A affinity chromatography using HiTrap Mabselect SuRe columns.

### 2.4. Mutagenesis of Biosimilar rAb Generation

A mutagenized heavy chain gene containing tryptophan to valine substitution within the CH2 domain of the heavy chain (at position C41 by IMGT codon numbering or 290 by Kabat numbering) was synthesized and cloned into the expression vector containing the light chain gene. The newly cloned DNA was transfected into CHO-k1 cells and mutagenized rAb was purified from cell supernatant using HiTrap Mabselect SuRe columns.

### 2.5. Stable Expression of the Biosimilar rAbs after Cell Line Generation

Cell lines for the biosimilar rAbs were generated using suspension-adapted CHO-k1 cells. CHO-k1 cells were preadapted in serum-free suspension culture and maintained in CDM4CHO (Hyclone, Little Chalfont, UK) chemically defined medium. 20 *μ*g of the wild-type expression vector was used to transfect 2 × 10^6^ cells by electroporation following a proprietary recombination-based transfection protocol. Transfected cells were monitored daily and subject to media change and antibiotic selection with puromycin (8 ug/mL) every 48 hours for 12-14 days. Stably transfected cell pool was then used for the selection of high titer cell lines by FACS cell sorting using MoFlo-XDP (Beckman Coulter, Brea, CA, USA). FACS cell sorting was performed after staining the cells using R-PE conjugated F(ab')_2_ fragment goat anti-human IgG Fc*γ* fragment specific antibody (Jackson ImmunoResearch Laboratories, Inc., West Grove, PA, USA) following the protocol in Brezinsky et al. (2003) [23]. High titer cell lines were sorted directly into 96-well plates containing 200 *μ*l of proprietary single cell growth medium per well, based on the R-PE fluorescence expression levels, and after excluding dead cells and doublets. Cell sorting was performed using the single cell sort mode to increase the efficiency sorting one cell per well. Cells sorted into 96 well plates were monitored for cell growth and clonality by scanning the plate using Clone Select Imager (Molecular Devices, San Jose, CA, USA). Approximately 15 days after the sorting, expression levels of the biosimilar rAb were determined with an ELISA assay and 20 clones with the highest expression levels were expanded for further analysis. A final clone with the highest cell titer was selected from the 20 clones, preserved in liquid nitrogen stocks, and then used for the expression of the biosimilar in a fed-batch condition. For the expression of the biosimilar from the cell line, cells were seeded in 30 ml of CDM4CHO with 8 *μ*g/ml puromycin and 800 *μ*l of Sheff Pulse II (Kerry Bioscience, Ithaca, NY, USA) feeding medium was added to the culture every 48 hours. Cells were maintained at 32°C for 10-12 days and harvested when cell viability dropped below 75%. Biosimilar rAb was purified from the cell culture supernatant using HiTrap Mabselect SuRe protein A columns.

### 2.6. Protein Quantitation with Indirect ELISA Assay

96-well plates for ELISA assay were prepared by coating the wells with goat anti-human IgG whole molecule primary antibody (5 *μ*g/mL, Sigma Aldrich) and blocking with tris-buffered saline with BSA pH 8. Purified IgG from human serum was used as a standard in serial dilutions in two folds starting from 50 ng/ ml. Biosimilar rAb samples were diluted so that the titer estimation can be made within the standard curve range. Standards and samples were added to the wells in duplicates. After extensive washing, the wells were probed with goat anti-human IgG- (gamma-chain specific-) peroxidase antibody (1:20,000, Sigma Aldrich) and colorized with 3,3′,5,5′-Tetramethylbenzidine (TMB) liquid substrate. Color change was induced with 1% HCl and the absorbance values were measured at 450 nm with VMax microplate reader (Molecular Devices).

### 2.7. Protein Characterization with SDS-PAGE and Western Blotting

The protein was characterized with sodium dodecyl sulfate polyacrylamide gel electrophoresis (SDS-PAGE) and Western blotting. Samples were reduced in a 1x sample reducing agent at 90-10°C for 5 min. The samples, along with a protein standard, were loaded into a 4-20% Tris-PAG precast gel (Komabiotech, Seoul, Korea) with 1x Tris-Glycine SDS running buffer for 90 min at a constant voltage of 120V. The resolved protein was either stained using Coomassie blue reagent overnight and destained with 50% ddH_2_O, 40% methanol and 10% acetic acid before imaging or transferred to a nitrocellulose membrane using the TransBlot-Semidry Transfer System (Bio-rad) for the Western blotting. The membranes were blocked in 5% NFDM and incubated in a primary antibody solution containing goat anti-human IgG whole molecule antibody (1:100,000, Sigma Aldrich). The membrane was probed with rabbit anti-goat IgG HRP-conjugated antibody (1:20,000, Sigma Aldrich) and detected using a LumiGLO chemiluminescent substrate kit (KPL). The relative intensity of the protein bands was scanned using FusionFx (Vilber, Collegien, France).

### 2.8. In-Solution Enzymatic Digestion

100 ug of protein sample obtained from protein A affinity chromatography was denatured with 50 mM ammonium bicarbonate (ABC) and 2 M urea. The sample was reduced by 100 mM DTT for 1 h at 35°C. After incubation, the sample was alkylated with 100 mM IAA for 1 h in a darkroom and digested with trypsin (0.125 ug/uL) overnight at 37°C. Digested sample was dried in a SpeedVac and stored in −20°C or reconstituted in 1% FA for desalting. SPE micro-spin column was equilibrated by centrifugation with wash buffer (99% H_2_O, 1% FA) and elution buffer (80% ACN, 1% FA). Aliquot of the tryptic digests was applied to the column, washed twice, and eluted twice with 30 uL elution buffer. The eluted peptide sample was dried in a SpeedVac.

### 2.9. In-Gel Enzymatic Digestion

In-gel protein sample excised from SDS-PAGE was repeatedly dehydrated with 500 uL ACN for 10 min at room temperature. The gel slices were reduced with 75 uL DTT for 30 min at 56°C, alkylated with IAA for 20 min in a darkroom, and destained with 500 uL 100 mM ammonium bicarbonate/acetonitrile (ABC/ACN) for 30 min with periodic vortexing. After dehydration, the gel slices were rehydrated with 10 mM ABC and 10% ACN buffer containing trypsin and digested at 37°C overnight. 5% FA/ACN (1:2, vol/vol) was added to the digested mixture and incubated at 37°C for 15 min with slight vortex. The digested sample was dried in a SpeedVac and either stored in −20°C or reconstituted in 0.1% FA for MS/MS analysis. Aliquot of the tryptic digests was applied to SPE micro-spin column, washed twice, and eluted twice with 30 uL elution buffer. The eluted peptide sample was dried in a SpeedVac.

### 2.10. LC-ESI-MS/MS Analysis

Tryptic peptide sample reconstituted with water containing 0.1% formic acid was separated by a Nano Acquity UPLC system (Waters, USA). 5 uL aliquot of the peptide solution was applied to a C_18_ trap column (i.d. 180 um, length 20 mm, and particle size 5 um; Waters, USA) with an autosampler. The peptides were desalted in the trap column for 10 min at a 5 uL/min flow rate. The trapped peptides were back-flushed into a homemade C_18_ trap column (i.d. 100 um, length 200 mm, and particle size 3 um) for separation. Mobile phases A and B were composed with 100% H_2_O containing 0.1% FA and 100% ACN containing 0.1% FA, respectively. The LC gradient began at 5% mobile phase B and was maintained for 15 min. The gradient was ramped up to 15% B for 5 min, 50% B for 75 min, and 95% B for 1 min. 95% B was maintained for 13 min and decreased to 5% B for 1 min. The column was finally re-equilibrated with 5% B for 10 min. LTQ Orbitrap Elite mass spectrometer (Thermo Fisher Scientific) equipped with a nanoelectrospray source was used to analyze the separated peptides. The electrospray voltage was set to 2.2 kV and the LTQ Orbitrap Elite mass spectrometer (Thermo Fisher Scientific) was operated in data-dependent mode during the chromatographic separation. The MS acquisition parameter for full-scan resolution was 60,000 in the Orbitrap for each sample. Data-dependent MS/MS scans were acquired by collision-induced dissociation (CID) and higher-energy collision dissociation (HCD). CID and HCD fragmentation scans were acquired in linear trap quadrupole (LTQ) mode with a 30-ms activation time and in Orbitrap at resolution 15,000 with a 20-ms activation time. A 35% normalized collision energy (NCE) and 5.0 Da isolation window were used for CID and HCD analyses. Previously fragmented ions were excluded for 300 s in all MS/MS scans. Raw mass data acquired were analyzed with the automated Glycoproteome Analyzer (GPA) algorithm to identify glycopeptides and then the resulting identified glycopeptides were further confirmed manually with careful investigation in retention time, chromatographic peak shape, isotopic mass distribution pattern, and fragmented ion matching.

## 3. Results and Discussion

### 3.1. LC-ESI-MS/MS Analysis of Reference rAb Glycoprotein

The reference rAb glycoprotein desalted with a HiTrap Mabselect SuRe protein A column (GE Healthcare) according to manufacturer's protocols was quantified by UV spectrometry, ELISA assay, and BCA assay and characterized by SDS-PAGE and Western blotting. The reference rAb glycoprotein sample desalted was reduced, alkylated in a darkroom, and digested with trypsin overnight. The digested sample was desalted with a SPE micro-spin column and analyzed by LC-ESI-MS/MS coupled with CID and HCD fragmentation techniques.

**Figure 1:**
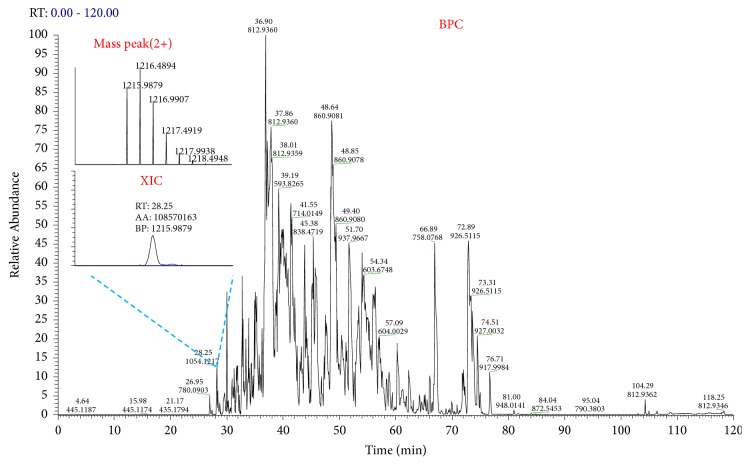
Base peak chromatogram (BPC) by LC-tandem MS analysis of the tryptic digests of reference rAb. The extracted ion chromatogram (XIC) of the glycopeptide EEQYNSTYR_3_3_1_0, identified as one of major components at RT 28.25 min, was also inserted together with the monoisotopic peak pattern of the glycopeptide.

**Figure 2:**
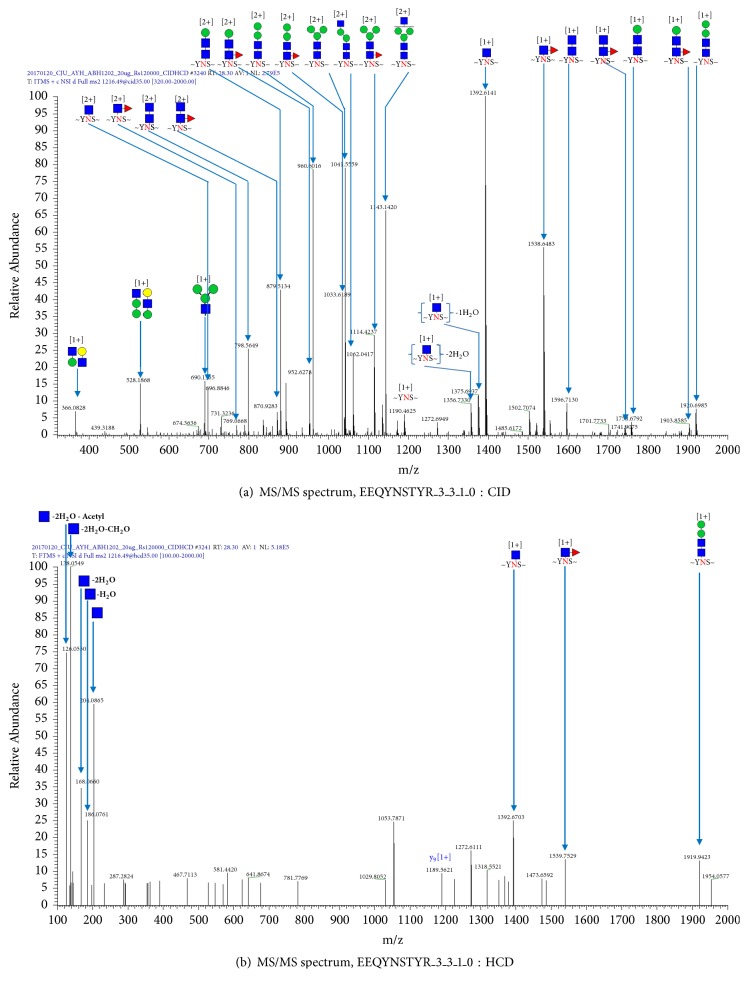
Tandem mass spectra of the glycopeptide EEQYNSTYR_3_3_1_0, obtained by (a) collision-induced dissociation and (b) higher-energy collision dissociation.

Figure 1 shows a base peak chromatogram (BPC) obtained from the analysis of the tryptic digests of the reference rAb glycoprotein. The obtained raw mass data was analyzed with GPA algorithm and the automatically assigned glycopeptides were further validated manually. Among the identified glycopeptides, EEQYNSTYR_3(hexose)_3(*N*-acetylhexosamine)_1(fucose)_0(sialic acid) was eluted at the retention time (RT), 28.25 min. The extracted ion chromatogram of the monoisotopic peak pattern of the precursor ion (2+) for glycopeptide EEQYNSTYR_3_3_1_0 was illustrated in Figure 1. Tandem mass spectra of the glycopeptide EEQYNSTYR_3_3_1_0, obtained by CID and HCD fragmentation techniques, were also shown in Figure 2. We confirmed that fragment ions were well-matched with their structures.

**Figure 3:**
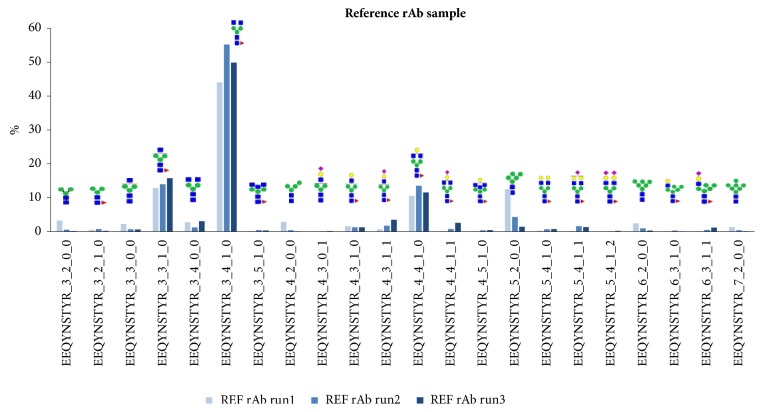
Triplicate analysis for glycosylation patterns of the reference rAb sample using LC-ESI-MS/MS. Each acquired mass dataset was normalized before comparative analysis. The sugar symbols: diamond (magenta) = sialic acid, circle (yellow) = galactose, square (blue) = GlcNAc, circle (green) = mannose, triangle (magenta) = fucose.

The analysis results of the reference rAb obtained by triplicate sample preparation and mass analyses were summarized in Table 1. LC-ESI-MS/MS with tryptic digestion detected 20 consistent glycosylation patterns with similar distributions. The glycosylation patterns normalized for each run were graphed together in Figure 3. We observed three major fucosylated glycopeptides, EEQYNSTYR_3_3_1_0, EEQYNSTYR_3_4_1_0, and EEQYNSTYR_4_4_1_0, which are reportedly predominant glycoforms found in recombinant antibodies produced in CHO cells [7, 24, 25]. In addition, high mannose forms with the relative ratios <13% were also observed—EEQYNSTYR_4_2_0_0, EEQYNSTYR_5_2_0_0, EEQYNSTYR_6_2_0_0, and EEQYNSTYR_7_2_0_0. These glycosylation patterns have been frequently observed in rAb therapeutics due to the fact that recombinant rAbs are not fully modified and substantially affected by posttranslational modifications [26, 27]. After confirming the reliability and repeatability in the analysis of the reference rAb glycoprotein, a comparative analysis was performed using biosimilar rAbs A and B, which were prepared by two different methods of production.

### 3.2. Comparative Analysis of Glycosylation Patterns in Biosimilar rAbs

**Figure 4:**
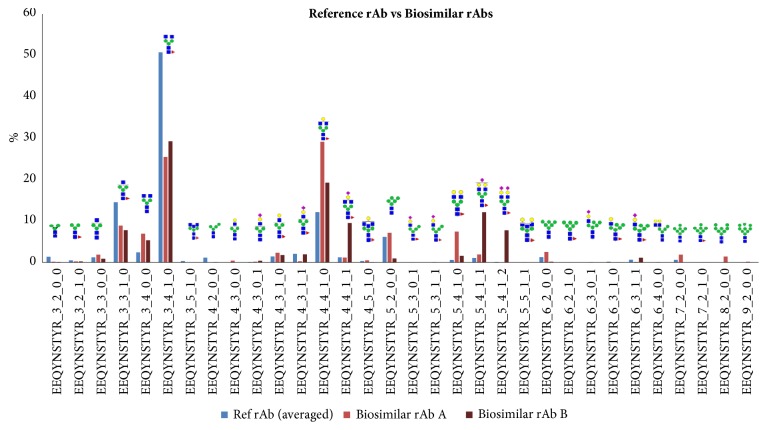
Comparison of glycosylation patterns of the reference rAb and the biosimilar rAb samples, obtained by LC-ESI-MS/MS. The glycosylation patterns for the reference rAb sample were obtained by triplicate analysis and averaged. All datasets acquired from each glycoprotein sample were normalized before comparative analysis.

Samples of biosimilar rAbs were obtained from either transiently transfected cells or a stably transfected cell line and referred to as biosimilar rAb A and B, respectively, for clarification purposes. The purified biosimilar rAbs A and B were digested with trypsin and analyzed with LC-ESI-MS/MS. The obtained raw mass data was analyzed with GPA algorithm and the identified glycopeptides were further validated manually. The glycosylation patterns acquired for the biosimilar rAb A and B were individually normalized and the mapping results were compared with those obtained from the analysis for the reference rAb sample (see Figure 4).

The major glycoforms for the biosimilar rAb A and rAb B as well as the reference rAb were consistent—EEQYNSTYR_3_3_1_0, EEQYNSTYR_3_4_1_0, and EEQYNSTYR_4_4_1_0. However, we observed variations in the minor glycosylation species not only between the reference rAb and biosimilar rAbs but also between the two biosimilar rAbs samples produced using different methods. The biosimilar rAb A and B displayed a decreased abundance for EEQYNSTYR_3_4_1_0 and an increased abundance for EEQYNSTYR_4_4_1_0 with the addition of galactose, in comparison to the reference rAb. The biosimilar rAb B displayed higher abundance for glycans with sialic acids—EEQYNSTYR_4_4_1_1, EEQYNSTYR_5_4_1_1, and EEQYNSTYR_5_4_1_2—in comparison to the biosimilar rAb A. These results suggest that LC-tandem mass spectrometry can be a highly sensitive tool for distinguishing subtle differences in minor glycosylation species.

### 3.3. Glycosylation Patterns in Mutagenized rAb Glycoprotein

**Figure 5:**
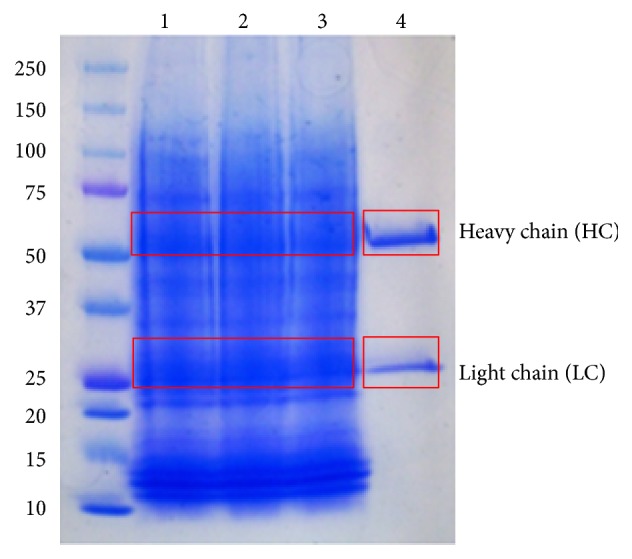
SDS-PAGE result for mutagenized rAb samples (lanes 1-3) and reference rAb glycoprotein (lane 4). Heavy chain and light chain bands were observed at 51 kDa and 26 kDa, respectively.

We tested the potential validity of using LC-tandem mass spectrometry in the determination process for the similarity of biosimilar products to the reference materials in biosimilar samples with a single mutation in amino acid sequences. Unintentional mutations that arise during protein production can cause changes in glycosylation patterns, which in turn can impact the safety, quality, and potency of the recombinant rAbs [28]. A tryptophan to valine amino acid mutation was created in the vicinity of the glycosylation site of Adalimumab to test its effect on the pattern of glycosylation to be detected using LC-tandem mass spectrometry. Interestingly, the single amino acid mutation caused the inability for biosimilar rAb to bind to the protein A affinity column (data not shown). So, instead of purifying the mutagenized rAb using protein A columns, we performed the analysis using in-gel samples. Unpurified samples were analyzed by SDS-PAGE and the expected heavy chain and light chain bands were observed at 51 kDa and 26 kDa, respectively (see Figure 5). The bands excised from the gel were subjected to in-gel tryptic digestion. The digested sample was desalted by ZipTip for mass spectrometry.

**Figure 6:**
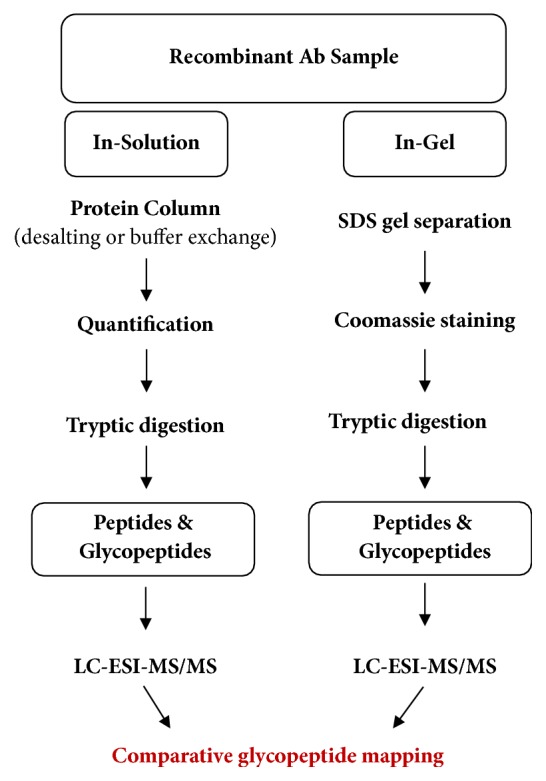
Comparative analysis for in-solution and in-gel rAb samples. In-solution sample is applied to a protein column and subject to tryptic digestion. In-gel sample is separated from SDS-PAGE gel and destained before tryptic digestion.

**Figure 7:**
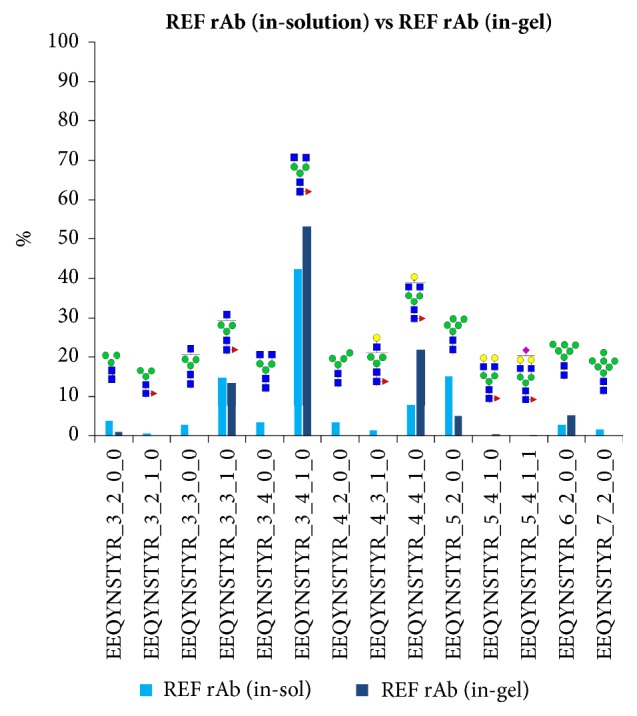
Comparison of the glycosylation patterns of the reference rAb, obtained, respectively, from the in-solution and the in-gel method. The two methods for sample preparation provided comparable results, implying that the in-gel method can be complimentary to the in-solution method for mapping protein glycosylation for our purposes.

Prior to analysis of the tryptic digests of the mutagenized rAb glycoprotein, a comparative analysis was performed between the in-solution reference rAb and the in-gel reference rAb glycoprotein to ensure the reliability and repeatability of the developed glycopeptide mapping methods (see Figure 6). The results in Figure 7 show that the mass spectrometry analysis of both the in-solution and the in-gel reference rAb samples yielded similar results with consistent glycosylation patterns, proving the reliability of the method.

**Figure 8:**
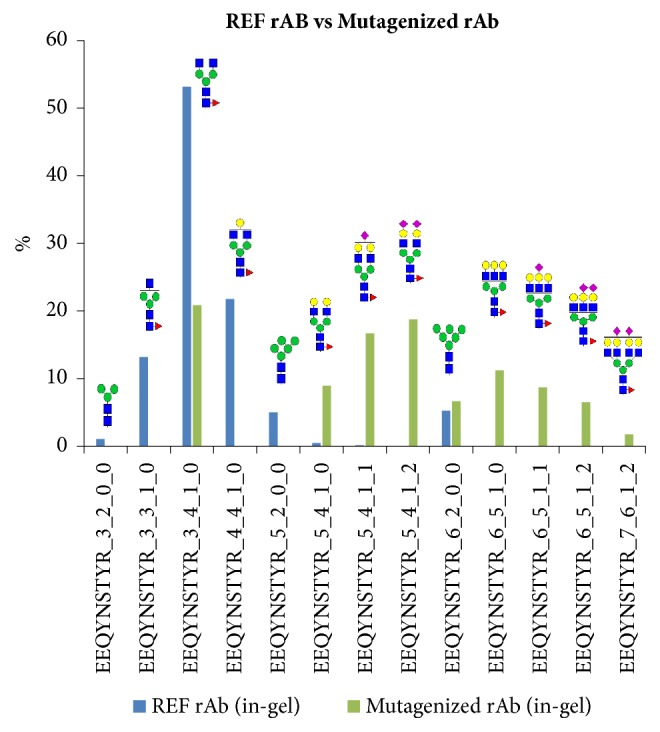
Comparative glycopeptide mapping of the reference rAb and the mutagenized rAb glycoprotein by in-gel digestion and LC-ESI-MS/MS. Each dataset was normalized before comparative analysis. Glycosylation patterns were notably different between the reference rAb and the mutagenized rAb samples. Increased abundance of sialylation, galactosylation, and branching was observed in the mutagenized rAb in comparison to the reference rAb glycoprotein.

We then conducted the analysis of the in-gel tryptic digests of the mutagenized rAb glycoprotein and compared its pattern against the in-gel tryptic digests of the reference rAb glycoprotein. As shown in Figure 8, we observed more substantiated variations in overall glycosylation patterns. Again, the major glycoforms (including EEQYNSTYR_3_3_1_0, EEQYNSTYR_3_4_1_0, and EEQYNSTYR_4_4_1_0) maintain similarity between samples to a certain extent, consistent with the previous analysis results of the reference rAb and the wild-type biosimilar samples prepared by the in-solution method. On the other hand, the differences in glycosylation patterns in minor species of the mutagenized rAb were more substantiated in comparison to those of the reference rAb. The mapping of the mutagenized rAb glycoprotein displayed highly sialylated and galactosylated glycoforms, such as EEQYNSTYR_5_4_1_1, EEQYNSTYR_5_4_1_2, EEQYNSTYR_6_5_1_1, and EEQYNSTYR_6_5_1_2. We also observed the increase in high-branched glycoforms, such as EEQYNSTYR_6_5_1_0, EEQYNSTYR_6_5_1_1, EEQYNSTYR_6_5_1_2, and EEQYNSTYR_7_6_1_2. Overall, we observed a significant increase of sialylated, galactosylated, and branched glycoforms in the mutagenized rAb glycoprotein. These substantial differences in glycosylation patterns can be attributable to the single amino acid mutagenesis and are as expected from the observation that the single amino acid change also caused the rAb's inability to bind to protein A column.

## 4. Conclusions

The replicate analyses of the reference rAb glycoprotein allowed us to conclude that the comparative glycopeptide mapping method using LC-tandem mass spectrometry and automated Glycoproteome Analyzer (GPA) software is a highly robust method for monitoring glycosylation patterns. When applying this comparative mapping method to the analyses of the reference and biosimilar rAbs, variations in the distribution of galactosylated and sialylated glycoforms in minor glycoform species were detected without any significant changes in the major glycoform species. These variations on the minor glycoform species of the biosimilar rAb compared to the reference rAb likely reflect the inherent nature of variations in biosimilar development, such as variations in cell lines used and various conditions for culturing the cells in bioreactors for the production of biosimilars. The ability of LC-tandem mass spectrometry-based system to readily detect such delicate variations in minor glycoform species suggests that the method of analysis can be an effective tool to be used in determining similarities of biosimilars to the reference materials in terms of glycosylation patterns with increased accuracy and possibly supplementing results obtained from the more widely used 2AB-based methods. It is not clear at this point, however, if these subtle variations in the minor glycoform species without any significant variations on the major glycoform species can become the basis for the failure of the biosimilar rAb in proving similarity with the reference rAb within the scope of the FDA guideline. The possible variations in functionality and/or immunogenicity of the biosimilar rAb over the reference rAb can only be determined with additional tests following this report.

We also tested the effect of a single amino acid mutation on the overall pattern of glycosylation of the biosimilar rAb. Coincidently, this single amino acid substitution introduced within the CH2 domain of the heavy chain (at position C41 by IMGT codon numbering or 290 by Kabat numbering) of IgG molecule caused the inability of the mutagenized biosimilar rAb to bind to protein A column. Without a readily available method to purify the mutagenized biosimilar rAb, we used in-gel based analysis of the glycoproteins. The in-gel based method was first proven to be effective when compared with in-solution based method in the analyses performed using the reference rAb sample. The in-gel based analysis of the mutagenized rAb glycoprotein showed that a single amino acid mutation in the vicinity of the IgG Fc glycosylation site can cause an increased level of variations on the overall pattern of glycoforms and with more pronounced variations on the minor species. The fact that a single mutation in the amino acid sequence of biosimilar can cause a significant change in the resulting glycosylation patterns and/or physiochemical properties emphasizes the importance of detailed glycopeptide mapping methods, such as LC-tandem mass spectrometry-based method as described our experiment, which can distinguish even slight variations on the minor glycoform species as an added accuracy in biosimilar development.

## Figures and Tables

**Table 1:** List of tryptic glycopeptides from triplicate analysis of reference rAb^a^.

No.	Glycopeptides	Ion Charge (+)	Detected Mass (m/z)	Run 1		Run 2		Run 3	
				RT (min)	MS_Intensity	RT (min)	MS_Intensity	RT (min)	MS_Intensity
1	EEQYNSTYR_3_3_0_0	2	1142.959	27.70	92380184	28.39	3270910	28.40	10319768
2	EEQYNSTYR_3_3_1_0	2	1215.989	27.41	372193798	28.31	72807858	28.30	188794868
		3	810.996						
3	EEQYNSTYR_3_4_0_0	2	1244.499	27.70	91063963	28.39	15496872	28.42	15407082
		3	830.000						
4	EEQYNSTYR_3_4_1_0	2	1318.029	27.40	1541540913	28.28	398363214	28.30	815484054
		3	878.688						
5	EEQYNSTYR_3_5_1_0	3	946.380	27.60	7447300	28.32	5456578	28.30	7199469
6	EEQYNSTYR_4_2_0_0	2	1122.443	27.60	126933115	28.39	1113753	28.35	6721632
7	EEQYNSTYR_4_3_0_1	3	913.355	28.28	188888	28.88	2653427	28.90	1300713
8	EEQYNSTYR_4_3_1_0	2	1297.018	27.40	50607743	28.25	6312278	28.21	20746171
9	EEQYNSTYR_4_3_1_1	2	1442.557	28.10	29760350	28.81	55607576	28.80	28356277
		3	962.043						
10	EEQYNSTYR_4_4_1_0	2	1398.554	27.38	285767417	28.25	58136525	28.20	205614717
		3	932.706						
11	EEQYNSTYR_4_4_1_1	3	1029.738	28.10	5127014	28.80	36633653	28.85	12664730
12	EEQYNSTYR_4_5_1_0	3	1000.398	27.54	2426554	28.32	4640770	28.30	6085787
13	EEQYNSTYR_5_2_0_0	2	1203.473	27.50	542316083	28.28	16939021	28.29	69462798
		3	802.652						
14	EEQYNSTYR_5_4_1_0	3	986.723	27.36	3496656	28.10	2860860	28.10	10937706
15	EEQYNSTYR_5_4_1_1	3	1083.755	28.10	8174096	28.81	18861846	28.80	25603836
16	EEQYNSTYR_5_4_1_2	3	1180.784	28.52	1129042	29.20	3397473	29.29	1181060
17	EEQYNSTYR_6_2_0_0	2	1284.497	27.40	102132925	28.18	733668	28.21	14024518
18	EEQYNSTYR_6_3_1_0	3	973.049	27.36	4256849	28.18	638835	28.20	4721699
19	EEQYNSTYR_6_3_1_1	3	1070.081	28.04	5703122	28.74	18933930	28.70	8336957
20	EEQYNSTYR_7_2_0_0	2	1365.524	27.40	55356342	28.18	559700	28.21	5262829

^a^MS_Intensity is based on the summation of 3-top monoisotopic mass peak intensity [21]. If simultaneously detected, MS_Intensity values of 2^+^ and 3^+^ mass ions are summed.

## Data Availability

The data used to support the findings of this study are available from the corresponding author upon request.
